# Tamoxifen Isomers and Metabolites Exhibit Distinct Affinity and Activity at Cannabinoid Receptors: Potential Scaffold for Drug Development

**DOI:** 10.1371/journal.pone.0167240

**Published:** 2016-12-09

**Authors:** Benjamin M. Ford, Lirit N. Franks, Anna Radominska-Pandya, Paul L. Prather

**Affiliations:** 1 Department of Pharmacology and Toxicology, College of Medicine, University of Arkansas for Medical Sciences, Little Rock, AR, United States of America; 2 Department of Biochemistry and Molecular Biology, College of Medicine, University of Arkansas for Medical Sciences, Little Rock, AR, United States of America; University of North Dakota, UNITED STATES

## Abstract

Tamoxifen (Tam) is a selective estrogen receptor (ER) modulator (SERM) that is an essential drug to treat ER-positive breast cancer. Aside from known actions at ERs, recent studies have suggested that some SERMs like Tam also exhibit novel activity at cannabinoid subtype 1 and 2 receptors (CB1R and CB2Rs). Interestingly, *cis-* (E-Tam) and *trans-* (Z-Tam) isomers of Tam exhibit over a 100-fold difference in affinity for ERs. Therefore, the current study assessed individual isomers of Tam and subsequent cytochrome P450 metabolic products, 4-hydroxytamoxifen (4OHT) and 4-hydroxy-N-desmethyl tamoxifen (End) for affinity and activity at CBRs. Results showed that Z-4OHT, but not Z-Tam or Z-End, exhibits higher affinity for both CB1 and CB2Rs relative to the E-isomer. Furthermore, Z- and E-isomers of Tam and 4OHT show slightly higher affinity for CB2Rs, while both End isomers are relatively CB1R-selective. When functional activity was assessed by G-protein activation and regulation of the downstream effector adenylyl cyclase, all isomers examined act as full CB1 and CB2R inverse agonists. Interestingly, Z-Tam appears to be more efficacious than the full inverse agonist AM630 at CB2Rs, while both Z-Tam and Z-End exhibit characteristics of insurmountable antagonism at CB1 and CB2Rs, respectively. Collectively, these results suggest that the SERMs Tam, 4OHT and End elicit ER-independent actions via CBRs in an isomer-specific manner. As such, this novel structural scaffold might be used to develop therapeutically useful drugs for treatment of a variety of diseases mediated via CBRs.

## Introduction

Cannabinoid receptors (CBRs) are seven-transmembrane spanning G-protein coupled receptors that occur as two subtypes sharing little homology, cannabinoid 1 receptor (CB1R) and cannabinoid 2 receptor (CB2R) [1]. CB1Rs are ubiquitously expressed in the CNS and are targets for the endogenously produced cannabinoids (*e*.*g*., endocannabinoids) 2-arachidonylglycerol (2-AG) and anandamide (AEA) [2]. Also modulated by endocannabinoids are CB2Rs, found primarily on immune cells such as T cells and macrophages and their activation produces anti-inflammatory and antinociceptive effects [3]. Both CBR subtypes modulate G_i/o_ proteins to produce downstream intracellular effects via inhibition of adenylyl cyclase activity, opening of inward rectifying K^+^ channels, and closing of voltage-gated Ca^2+^ channels [4, 5].

Although potential therapeutic uses for drugs acting via CBRs have been sought for decades, drug development in this area has been significantly limited by potential abuse liability and psychotropic effects produced by activation of CB1 receptors in the CNS by compounds such as Δ^9^-tetrahydrocannabinol (Δ^9^-THC) present in marijuana (*Cannabis sativa)* and synthetic cannabinoids found in the emerging drugs of abuse known as K2 and spice [6, 7]. Despite such potential adverse effects, CBRs remain therapeutic targets for development of drugs to treat a diverse range of diseases including cancer, obesity, chronic pain, alcohol abuse, osteoporosis, nausea and peripheral tissue injury [7–11].

Development of therapeutic drugs acting via CBRs is promising not only because of important roles that endocannabinoids play in many disease states, but also due to the structural diversity of drugs that have been found to bind and modulate the activity of CBRs. As such, identifying novel structural scaffolds to develop potent and efficacious CBR agonists, antagonists and/or inverse agonists is being vigorously pursued by several groups [12–15]. However, due to the adverse effects of currently available drugs acting at CBRs, FDA approval of therapeutic cannabinoids unfortunately remains elusive. Recent studies by our group [16] and others [17, 18] have shown that several clinically available, FDA-approved drugs in the selective estrogen receptor modular (SERM) class (e.g. Z-Tamoxifen, Z-4-hydroxytamoxifen, and Raloxifen) also bind and modulate activity of CB1 and CB2Rs. SERMs exhibit few adverse effects and characterization of their actions at CBRs is lacking. Therefore, detailed studies are needed to determine if novel drugs acting via CBRs, derived from the SERM scaffold, might offer distinct advantages relative to currently available cannabinoids.

**Fig 1:**
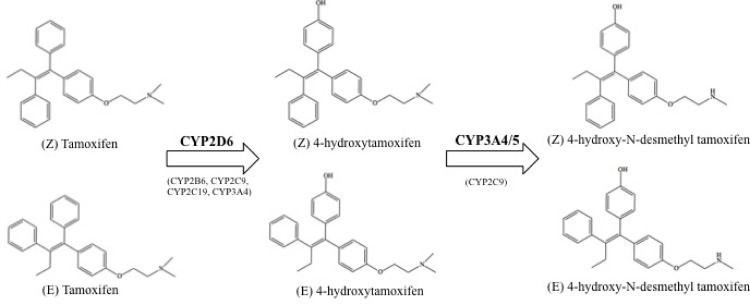
Structure of tamoxifen stereoisomers and subsequent cytochrome P450 metabolites. E- and Z-Tamoxifen (Tam) are metabolized by several CYP450 enzymes, the primary being CYP2D6 and CYP3A4/5, which yields stereoisomers of 4-hydroxytamoxifen (4OHT) and 4-hydroxy-N-desmethyl tamoxifen (End), respectively [33].

Tamoxifen (Tam) is a well-known SERM that has served as a mainstay for treatment of ER-positive breast cancer [19, 20]. Upon administration, Tam acts as a pro-drug, and via cytochrome P450 metabolism to 4-hydroxytamoxifen (4OHT) and 4-hydroxy-N-desmethyltamoxifen (End; Fig 1), leads to potent antagonism of ERs and inhibition of estrogen-responsive gene transcription [21, 22]. Because Tam, 4OHT and End each contain a double bond, *cis*- (E) and *trans*- (Z) isomers are formed that possess remarkably different binding affinities and effects at ERs. For example, Z-Tam binds to ERs with a 100-fold greater affinity than E-Tam. Functionally, Z-Tam acts as an ER antagonist, while E-Tam acts as an ER agonist [23, 24]. Similar differences in affinity and intrinsic activity favoring the Z isomer have also been observed with 4OHT and End [25, 26]. Such distinct modulation of ERs by the E and Z isomers of Tam, 4OHT and End [27] suggest that these isomers might also exhibit novel affinity and activity at CB1 and CB2Rs.

This study was designed to test the hypothesis that the E and Z isomers of Tam, 4OHT, and End exhibit distinct affinity and activity at CB1 and CB2Rs. To achieve this goal, the affinity of SERMs for CBRs was determined by competition binding studies employing CHO cells stably transfected with human CB1 and CB2Rs. CBR activity was also assessed in transfected CHO cells by assessing the ability of SERMs to modulate G-protein activity and regulate activity of the downstream intracellular effector adenylyl cyclase. Identification of high affinity SERMs that modulate CBRs in an isomer-selective manner would suggest that this novel structural scaffold might be employed for development of safe and efficacious drugs acting at CBRs for a variety of diseases mediated via CBRs.

## Methods

### Materials

The following SERMs were purchased from the commercial sources as indicated: E-Tam from Carbosynth (San Diego, CA), Z-Tam from Cayman Chemical (Ann Arbor, MI), E-4OHT from Tocris Bioscience (Ellisville, MO), Z-4OHT from Sigma-Adrich (St. Louis, MO), E-End from Santa Cruz Biotechnology, Inc. (Dallas, TX), and Z-End from Axon Medchem (Reston, VA). WIN-55,212–2, CP-55,940, and DAMGO were obtained from Tocris Bioscience. GTPγS was procured from EMD Chemical (Gibbstown, NJ). [^3^H]CP-55,940 (131.4 Ci/mmol) was purchased from PerkinElmer (Waltham, MA) and [^35^S]GTPγS (1250 Ci/mmol) was obtained from American Radiolabeled Chemicals (St. Louis, MO). Pertussis toxin was acquired from List Biological Laboratories Inc. (Campbell, CA). All other reagents were purchased from Fisher Scientific (Pittsburgh, PA).

### Cell Culture assays

Chinese hamster ovary (CHO-K1) cells were stably transfected with the human cannabinoid subtype 2 receptor (CNR2; hCB2) [28] or the human mu-opiod receptor (MOR, CHO-hMOR) [29]. CHO cells stably expressing hCB1 receptors (CNR1; CHO-hCB1) were purchased from DiscoverRx Corporation (Fremont, CA). CHO-hCB2 and CHO-hMOR cell lines were cultured in DMEM (Mediatech Inc., Manassas, VA) while CHO-hCB1 cells were cultured in HAM’s F-12 K media (ATCC, Manssas, VA). Media for all cell types contained 10% fetal calf serum (Gemini Bioproducts, Sacramento, CA), 0.05 IU/mL penicillin, 50 μg/mL streptomycin (Invitrogen, Carlsbad, CA), and 250 μg/mL of Geneticin (or G418; Sigma-Aldrich, St. Louis, MO). All cell types were maintained in a humidified chamber at 37°C with 5% CO_2_, harvested when flasks reached approximately 80% confluency, and only cells from passages 1–15 were used in all experiments.

### Membrane Preparation

CHO-hCB1, CHO-hCB2 and CHO-hMOR cells were homogenized individually with 10 complete strokes utilizing a 7ml Dounce glass homogenizer in an ice-cold buffer containing 50 mM HEPES pH 7.4, 3 mM MgCl_2_, and 1 mM EDTA as described previously [13]. The homogenized samples were then centrifuged at 40,000 *× g* for 10 min at 4°C. Supernatants were discarded; the pellets re-suspended in the buffer, homogenized again, and centrifuged similarly twice more. After the final centrifugation step, supernatants were discarded and pellets were re-suspended in ice-cold 50 mM HEPES, pH 7.4 to achieve an approximate protein concentration of 10 mg/ml. Membrane homogenates were divided into aliquots and stored at −80°C for future use. A small aliquot of each membrane preparation was removed prior to freezing and the protein concentration was determined using BCA Protein Assay (Thermo Fisher Scientific, Waltham, MA).

### Competition Receptor Binding

Competition receptor binding was performed as reported earlier [30]. Briefly, each reaction mixture contained either 100 μg of CHO-hCB1-Rx or 50 μg of CHO-hCB2 membrane homogenates, 0.2 nM [^3^H]-CP55,940, 5 mM MgCl_2_, and increasing concentrations of the non-radioactive competing ligands in a 50 mM Tris-HCl buffer (pH 7.4) with 0.1% bovine serum albumin. The total volume of the incubation mixture was 1 ml. All reactions were mixed and allowed to reach equilibrium binding by incubation at room temperature for 90 min. Non-specific binding was defined as the amount of radioligand binding remaining in the presence of a 1 μM concentration of the non-radioactive, high affinity, CB1/CB2 agonist WIN-55,212–2. Binding was terminated by rapid vacuum filtration through glass fiber filters (Brandel, Gaithersburg, MD), followed by four 5 ml washes of ice-cold 50 mM Tris-HCl (pH 7.4) buffer containing 0.1% bovine serum albumin. Four ml of scintiverse scintillation fluid (Fisher Scientific, Waltham, MA) was added to the filters and the amount of radioactivity was quantified 24 hr later utilizing liquid scintillation spectrophotometry.

### [^35^S]GTPγS Binding

The GTPγS binding assay to measure G-protein activation was performed as previously described [30]. Briefly, in a total volume of 1 ml, 25 μg of CHO-hCB2, 50 μg of CHO-hCB1-Rx or 50 μg of CHO-hMOR membranes homogenates were added to each reaction mixture containing 0.1 nM [^35^S]GTPγS, 20 mM HEPES, 10 mM MgCl_2_, 100 mM NaCl, 10 μM GDP, 0.1% bovine serum albumin and the indicated concentrations of ligand to be examined. After mixing, reaction mixtures were incubated at 30°C for 30 min. (a time interval shown to produce optimal agonist-induced [^35^S]GTPγS binding levels, data not shown). Nonspecific binding was defined by the amount of radioactivity remaining in the presence of 10 μM non-radiolabeled GTPγS. Reactions were terminated by rapid vacuum filtration through glass fiber filters followed by four washes with ice cold 50 mM HEPES (pH 7.4) containing 0.1% bovine serum albumin. Four ml of scintiverse scintillation fluid (Fisher Scientific, Waltham, MA) was added to the filters and the amount of radioactivity was quantified 24 hr later utilizing liquid scintillation spectrophotometry.

### Measurement of Intracellular cAMP Levels in Intact Cells

CHO-hCB1, CHO-hCB2, or CHO-hMOR cells were plated separately in normal culture media into 24-well plates at a density of 6 × 10^6^ cells/plate and incubated overnight at 37°C in 5% CO_2_. As previously described [13], the following day culture media was removed from each well and replaced with 500 μl pre-incubation mixture containing either HAM’s F-12 K (CHO-hCB1) or DMEM (CHO-hCB2 and CHO-hMOR) with 0.9% NaCl, 500 μM 3-isobutyl-1-methylxathine (IBMX) and 2 μCi/well [^3^H]adenine. Cells were incubated for 3–5 hr at 37°C. The pre-incubation mix was removed and indicated concentrations of drugs were added to the individual wells for 15 min at 37°C in a Krebs-Ringer-HEPES solution (10 mM HEPES, 110mM NaCl, 25mM Glucose, 55mM Sucrose, 5mM KCl, 1mM MgCl_2_, 1.8 mM CaCl_2_, pH 7.4) containing IBMX, and 10 μM forskolin. Reactions were terminated by adding 50 μl 2.2N HCl and [^3^H]cAMP was isolated by employing alumina column chromatography. Radioactivity contained in 4 ml of the final column eluate was counted by a Packard-Tri-carb 2100/TR liquid scintillation counter after adding 10 ml of liquid scintillation cocktail.

To determine if modulation of adenylyl cyclase activity by SERMs was mediated through CB2 receptors coupling to G_i/o_ proteins, additional cAMP assays were conducted following overnight treatment with pertussis toxin as described elsewhere [28]. Briefly, CHO-hCB2 cells were seeded into 24-well plates as described above in normal culture media containing 100 ng/ml of pertussis toxin and incubated overnight at 37°C. Pertussis toxin-treated media was removed and measurement of intracellular [^3^H]cAMP levels following indicated SERM treatments was conducted as described above.

Experiments were also conducted to examine the ability of SERMs to antagonize the modulation of adenylyl cyclase activity by the CB1 agonist CP-55,940. After overnight seeding into 24-well plates and pre-incubation of cells with [^3^H]adenine for 3–5 hr at 37°C as described above, media was removed and the indicated concentration of the SERM to be tested was added to all wells of the 24-well plate. Plates were then incubated at room temperature for 30 min, followed by addition of increasing concentrations of CP-55,940 (10^−10^–10^−5^ M) and a final 7 min room temperature incubation. Reactions were terminated and [^3^H]cAMP isolated as described above.

### Statistical analyses

Data presented are expressed as mean ± standard error of the mean (S.E.M.) for a minimum of three experiments, each performed in triplicate. GraphPad Prism version 6.0f (GraphPad Software Inc.) was used for all curve-fitting and statistical analyses. Non-linear regression for one-site competition was used to determine the IC_50_ for competition receptor binding. IC_50_ values were subsequently converted to K_i_ values (a measure of receptor affinity) by the Cheng-Prusoff equation [31]. Non-linear regression was also used to analyze concentration-effect curves to determine the EC_50_ or IC_50_ (measures of potency) and E_max_ or I_max_ (measures of efficacy) for GTPγS binding and adenylyl cyclase experiments, respectively. All dissociation constants and measurements of potency were converted to pK_i_, pK_B_, pEC_50_, or pIC_50_ values by taking the negative log of each value so that parametric tests could be used for statistical comparisons. To compare three or more groups, statistical significance of the data was determined by a one-way ANOVA, followed by *post hoc* comparisons using a Tukey's or Dunnett's test. To compare two groups, the non-paired Student's *t*-test was used.

## Results

### SERM isomers exhibit distinct affinity and selectivity for hCB1 and hCB2Rs

**Fig 2:**
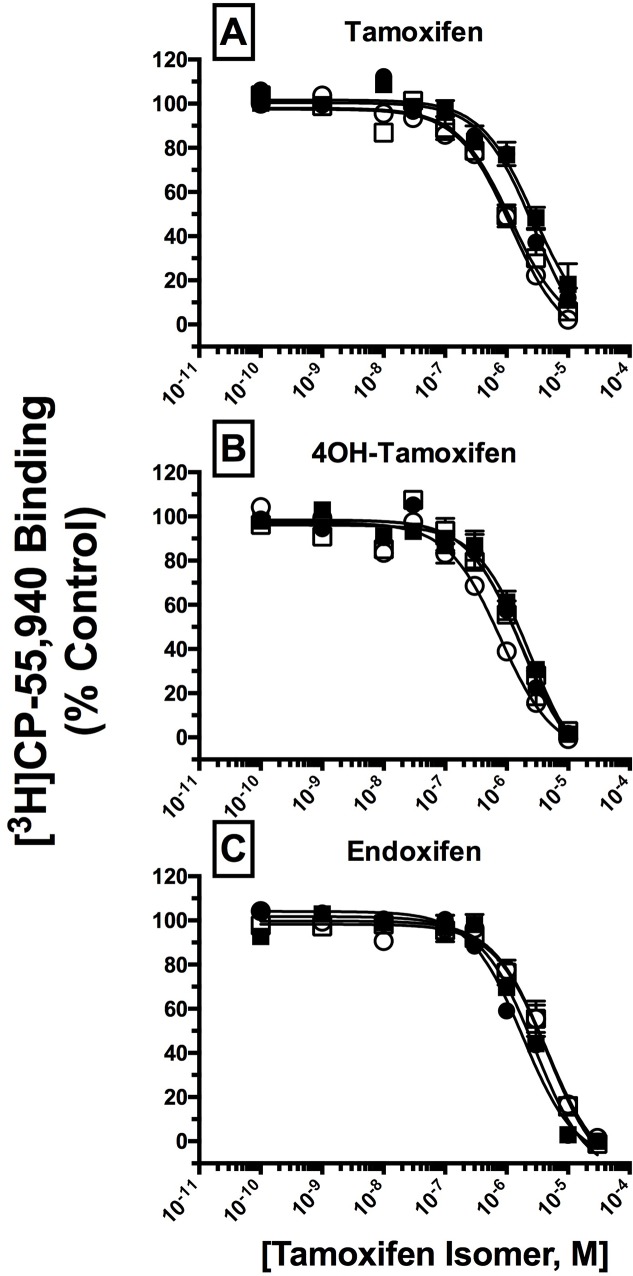
SERM isomers exhibit mid-nanomolar to low-micromolar affinities for CB1 and CB2Rs. A measure of affinity (K_i_) of E and Z isomers of Tam, 4OHT, and End for respective CB1 and CB2Rs was obtained by conducting competition binding studies, employing 0.2 nM [^3^H]-CP-55,940 and increasing concentrations of test compounds. K_i_ values (mean ± SEM) were derived from non-linear regression analysis of the curves shown in [A-C]. Individual K_i_ values and statistical analysis of pK_i_ values are presented in Table 1. Filled squares and circles represent binding of respective E and Z isomers to CB1Rs, open squares and circles represent binding of respective E and Z isomers to CB2Rs.

Initial competition binding studies, employing the high affinity CB1/CB2R agonist [^3^H]CP-55,940, were conducted to determine the affinity of E and Z isomers of Tam (Fig 2A), and its cytochrome P450-derived metabolites 4OHT (Fig 2B) and End (Fig 2C), for hCBRs. The affinities of all compounds for hCBRs are presented as K_i_ values (Table 1), derived from IC_50_ values [32] obtained from competition binding curves (Fig 2). K_i_ values were converted to pK_i_ values (pK_i_ = -Log[K_i_]; Table 1) so that parametric tests could be used for statistical comparisons. All compounds bound to hCB1Rs with affinities in the mid-nanomolar to low-micromolar range, with Z-4OHT exhibiting the highest affinity (681 nM) that was significantly different (P>0.05) from E- or Z-Tam. Concerning hCB2Rs, all compounds also exhibited mid-nanomolar to low-micromolar affinities; however, both E- and Z-End bound with significantly lower affinities (P>0.05) to hCB2Rs than isomers of either Tam or 4OHT. Interestingly, only the Z-isomer of 4OHT, but not isomers of Tam or End, exhibited higher affinity for both hCB1 (P<0.05) and hCB2 (P>0.01) receptors when compared to the E-isomer. Lastly, concerning CBR-selectivity of binding, both E- and Z-Tam bound with significantly higher affinity to hCB2Rs relative to hCB1Rs, with respective selectivity ratios (hCB1-K_i_/hCB2-K_i_) of 1.78 (P<0.05) and 1.97 (P<0.05). In marked contrast, Z-End exhibited significantly higher affinity for hCB1Rs, with a selectivity ratio of 0.49 (P<0.01). Both isomers of 4OHT and E-End bound to hCB1 and hCB2Rs with similar affinity. Collectively, these results suggest that isomers of Tam and its metabolites 4OHT and End exhibit subtle, but distinct, differences in affinity and selectivity for binding to hCB1 and hCB2Rs.

**Table 1:** Competition binding of SERM isomers employing CHO-hCB1 and CHO-hCB2 membranes.

Drug	[^3^H]CP-55,940 Binding						
	*CHO-hCB1*			*CHO-hCB2*			*Selectivity*
	K_i_ (nM)	pK_i_	N	K_i_ (nM)	pK_i_	N	(CB1/CB2)
**E-Tam**	**1510**	**5.821 ± 0.051**^**a**^	**5**	**847**	**6.072 ± 0.100**^**a**^	**3**	**1.78**^**†**^
**Z-Tam**	**1574**	**5.803 ± 0.081**^**a**^	**6**	**798**	**6.098 ± 0.071**^**a**^	**3**	**1.97**^**†**^
**E-4OHT**	**1242**	**5.906 ± 0.026**^**a**^^,^^**b**^^,^^*^	**3**	**957**	**6.019 ± 0.070**^**a**^^,^^*^	**3**	**1.30**
**Z-4OHT**	**681**	**6.167 ± 0.082**^**b**^	**3**	**495**	**6.305 ± 0.041**^**a**^	**3**	**1.38**
**E-End**	**1393**	**5.856 ± 0.047**^**a**^^,^^**b**^	**3**	**2355**	**5.628 ± 0.081**^**b**^	**3**	**0.59**
**Z-End**	**1161**	**5.935 ± 0.057**^**a**^^,^^**b**^	**3**	**2393**	**5.621 ± 0.021**^**b**^	**3**	**0.49**^**††**^

^a,b^pKi values designated by different letters are significantly different from values within the same column; P<0.05, one-way ANOVA, Tukey *Post-hoc* test.

*pKi values of the E-isomer are significantly different from the Z-isomer of the same compound within the same column; P<0.05, student’s *t*-test.

^†,††^ pKi values for hCB2 receptors are significantly different from hCB1 receptors; P<0.05, 0.01, student’s *t*-test.

### SERM isomers act as hCB1 and hCB2R inverse agonists to modulate G-protein activity

**Fig 3:**
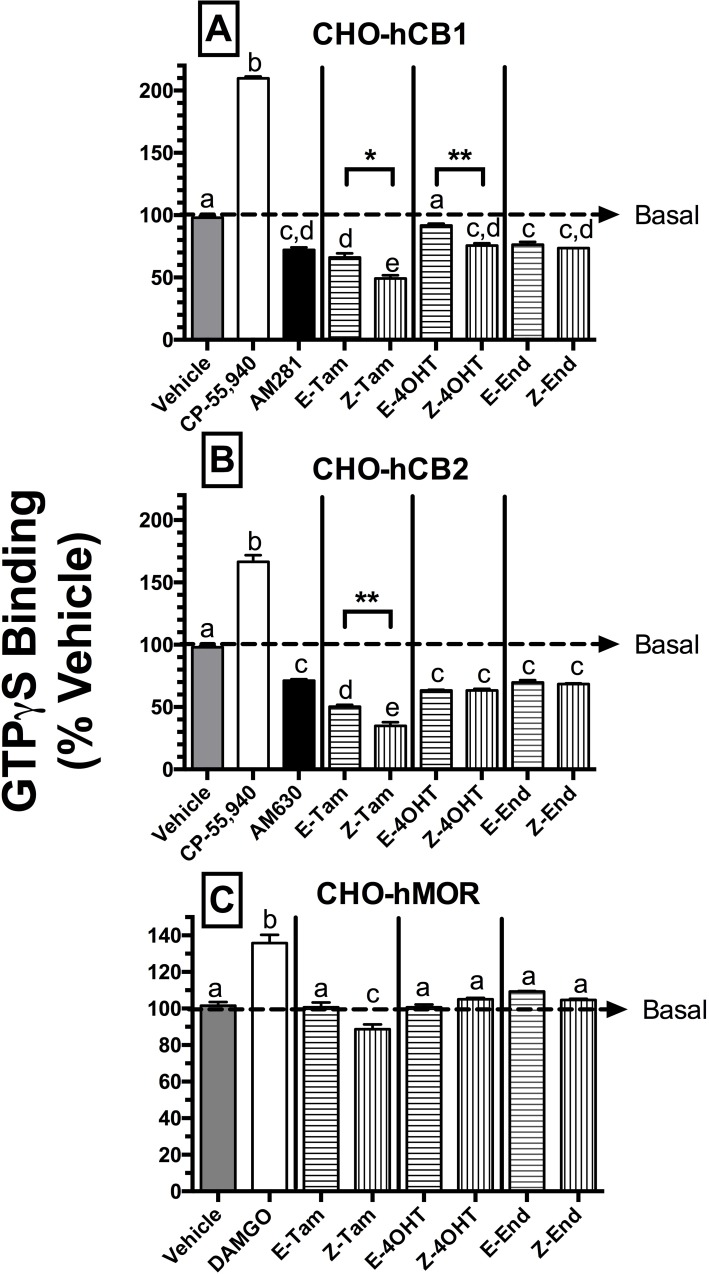
SERM isomers reduce basal G-protein activity via CB1 and CB2Rs. The ability of SERMs to modulate basal G-protein activity via [A] CB1R, [B] CB2R and [C] MORs was evaluated by examining [^35^S]-GTPγS binding in the presence or absence of a receptor-saturating concentration (10 μM) of all compounds. G-protein modulation by full agonists CP-55,940 (10 μM) and DAMGO (10 μM) was examined to serve as positive controls for activation of [A-B] CBRs and [C] MORs, respectively. G-protein modulation by the inverse agonists AM-281 and AM-630 was examined to serve as positive controls for regulation of [A] CB1 and [B] CB2R signaling. The mean ± SEM of [^35^S]GTPγS binding is presented as percent of G-protein activity in the presence of vehicle. ^a,b^[^35^S]GTPγS binding produced by individual SERMs acting at hCB1 [A], hCB2 [B] or hMOR [C] receptors designated by different letters above bars, is significantly different (P<0.05, one-way ANOVA; Tukey *Post-hoc* test). *,**Bar graphs comparing E and Z isomers of individual SERMs that are designated by asterisks, are significantly different from activity at respective receptors (P<0.05, 0.01; student’s t-test).

Since all SERMs examined were found to bind to hCB1 and hCB2Rs with moderate affinity, studies were next conducted to determine the intrinsic activity of these compounds by examining whether they act as agonists, antagonists or inverse agonists at hCBRs. Initial studies examined the ability of SERMs to modulate G-protein activity via hCBRs (Fig 3). CB1 and CB2Rs are G-protein coupled receptors that, upon ligand binding, modulate activity of G_i/o_*-*proteins [34, 35]. Binding of agonists to CBRs increase G_i/o_*-*protein activity, receptor interaction with neutral CBR antagonists does not alter the activity of G_i/o_*-*proteins and, because CBRs are constitutively active, inverse agonists reduce basal G_i/o_*-*protein activity. To measure G-protein activity, membranes prepared from CHO-hCB1 (Fig 3A), CHO-hCB2 (Fig 3B) or CHO-hMOR (Fig 3C) cells were incubated with the non-hydrolysable, radioactive GTP analog [^35^S]GTPγS, and a receptor saturating concentration (10 μM) of each SERM (*e*.*g*., >10 times K_i_; Table 1) that would be predicted to produce a maximal response. As anticipated [16, 29, 34], incubation with the known CB1R agonist CP-55,940 or CB1R inverse agonist AM-281 significantly increased or decreased [^35^S]GTPγS binding, respectively (Fig 3A). Consistent with actions as inverse agonists, all SERMs examined (except E-4OHT) reduced basal [^35^S]GTPγS binding to levels similar to that produced by the full CB1R inverse agonist AM-281 [36]. Interestingly, Z-Tam inhibited G-protein activity more efficaciously (P>0.05) than AM-281 (I_MAX_ = 50.8 ± 2.7% versus 33. ± 3.2%, respectively). In marked contrast, E-4OHT did not significantly alter basal [^35^S]GTPγS binding. Although this observation is consistent with actions of a neutral antagonist, future studies comparing E-Tam to an established CB1-selective neutral antagonist will be necessary to confirm whether or not E-Tam exhibits neutral antagonistic activity. SERMs also appear to act as full inverse agonists at hCB2Rs (Fig 3B), reducing basal [^35^S]GTPγS binding to levels similar to that produced by the known full CB2R inverse agonist AM-630 [34]. Importantly, both E- and Z-Tam produce a greater decrease (P<0.001) in basal G-protein activity than AM-630 (I_MAX_ = 49.7 ± 1.5%, 65.1± 3.0% and 28.7%± 1.1%, respectively). To confirm that effects of SERMs on G-protein activity observed result from specific interaction with hCBRs, similar studies were conducted in CHO cells devoid of hCBRs, but instead transfected with human mu-opioid receptors (CHO-hMOR; Fig 3C). As expected for the full hMOR agonist DAMGO [37], incubation of CHO-hMOR membranes with a receptor saturating concentration (1 μM) increased [^35^S]GTPγS binding. In marked contrast to previous results observed in CHO-hCB1 or CHO-hCB2 cells (Fig 3A and 3B), incubation of CHO-hMOR membranes with 10 μM of all SERMs (except Z-Tam) did not alter basal G-protein activity (Fig 3C). Although Z-Tam did significantly decrease [^35^S]GTPγS binding in CHO-hMOR membranes by 11.3% ± 2.6%, this small, presumably non-hCBR action in CHO cells might contribute the greater efficacy observed for this isomer relative to E-Tam observed at hCB1 (Fig 3A) and hCB2Rs (Fig 3B). In summary, these results suggest that isomers of Tam and its 4OHT and End metabolites act predominantly as full hCBR inverse agonists. However, similar to observations for receptor affinity, these novel compounds exhibit subtle, but distinct, differences in intrinsic activity at hCB1 and hCB2Rs.

### SERM isomers act as full hCB2R inverse agonists to modulate intracellular cAMP production

**Fig 4:**
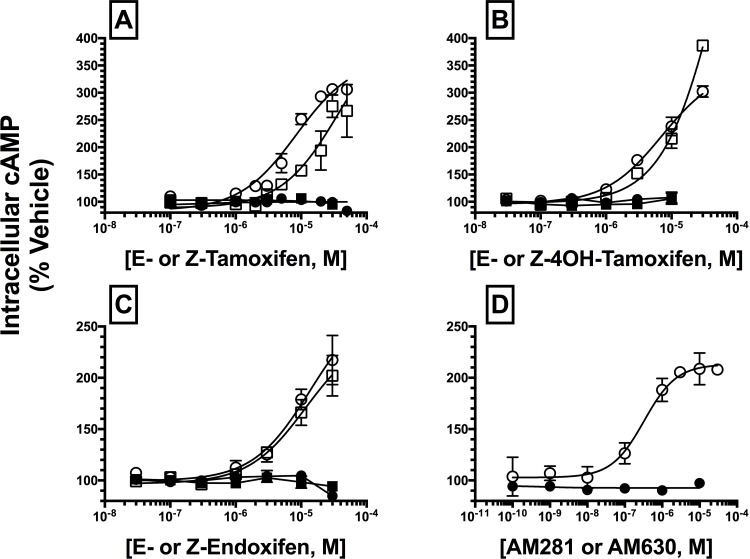
Modulation of forskolin-stimulated cAMP production by SERM isomers in intact CHO-hCB1 and CHO-hCB2 cells. The potency (IC_50_) and efficacy (E_MAX_) for modulation of forskolin-stimulated AC was evaluated by analyzing concentration-effect curves for SERMs in intact CHO-hCB1 and CHO-hCB2 cells. All IC_50_ and E_MAX_ values (mean ± SEM) were derived from non-linear regression analysis of the curves shown in [A-D] and are presented in Table 2 with statistical analysis. For panels [A-C], filled squares and circles represent modulation of adenylyl cyclase activity by E- and Z-isomers acting at hCB1Rs, respectively, while open squares and circles demonstrate modulation by E- and Z-isomers at CB2Rs. In panel [D], modulation of adenylyl cyclase activity by the selective CB1R inverse agonist AM-281 (filled circles) and CB2R inverse agonist AM-630 (open circles) is depicted.

**Fig 5:**
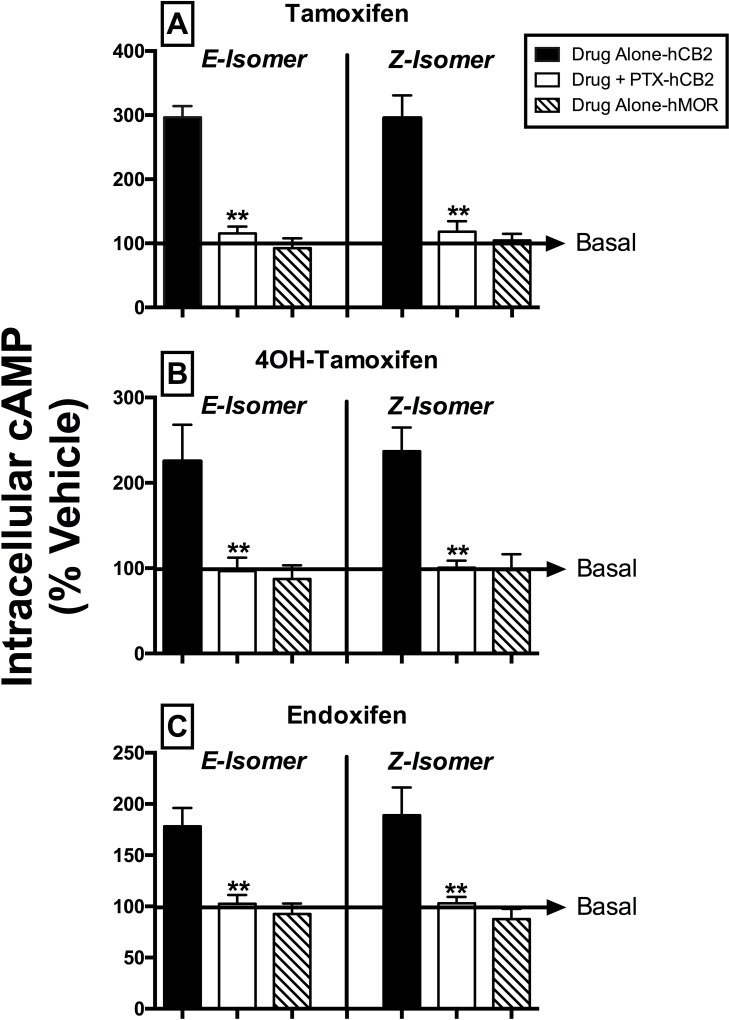
SERM isomers modulate forskolin-stimulated AC activation via G_i/o_ proteins and CB2Rs. [A-C] Modulation of forskolin-stimulated cAMP production by SERMs (10 μM) in intact CHO-hCB2 and CHO-hMOR cells was evaluated. Drugs were examined in CHO-hCB2 cells (+/- 100 ng PTX pretreatment) and in CHO-hMOR cells not expressing CBRs. Intracellular cAMP values (mean ± SEM) are presented as percent response compared to levels observed in the presence of vehicle. Statistics revealed that no drug altered basal cAMP levels in CHO-hCB2 cells treated with PTX (P<0.01; student’s *t*-test) or in CHO-hMOR cells (P<0.01; one-sample *t*-test).

To provide a second measure of intrinsic activity, experiments were next conducted to examine the ability of SERMs to modulate activity of the intracellular effector adenylyl cyclase via hCBRs in intact cells (Fig 4; Table 2). G_i/o_*-*proteins activated by CBRs proceed to regulate activity of the downstream intracellular effector adenylyl cyclase, resulting in alterations in cAMP levels [38]. Therefore, CBR agonists inhibit adenylyl cyclase activity to reduce intracellular cAMP levels, neutral CBR antagonists do not alter cAMP levels, and inverse agonists reduce constitutive activity of CBRs, resulting in an increase of cAMP levels. Full concentration-effect curves for the activity of all SERMs at both hCB1 (closed symbols) and hCB2Rs (open symbols) were conducted (Fig 4A–4D) and EC_50_ and E_MAX_ values were determined (Table 2). EC_50_ values were converted to pEC_50_ values (pEC_50_ = -Log[EC_50_]) so that parametric tests could be used for statistical comparisons. Curiously, unlike that observed for G-protein modulation, no SERM altered basal cAMP levels in CHO-hCB1 cells (Fig 4A–4C; closed symbols), indicative that in this assay these compounds could potentially act as neutral hCB1 antagonists. However, since the well characterized CB1R inverse agonist AM-281 also did not alter cAMP levels (Fig 4D; closed symbols), it is likely that the level of constitutive activity of hCB1Rs expressed in the CHO-hCB1 cell line employed here is apparently insufficient to detect inverse agonism when evaluated by this assay [36]. Therefore, based on these observations, no definitive conclusions can be made concerning the antagonist versus inverse agonist actions for CB1R-modulation of adenylyl cylcase activity by SERMs. Concerning hCB2 receptors, as anticipated, the full CB2R inverse agonist AM-630 produced potent, efficacious and dose-dependent increases in intracellular cAMP production (Fig 4D; open symbols). Similarly, all SERMs examined (Fig 4A–4C; open symbols) increased cAMP levels via hCB2Rs with similar potencies (*e*.*g*., EC_50_ values) in the low micromolar range, except for E-Tam that exhibited significantly lower potency (Table 2; P<0.01). When comparing individual isomers, the Z-isomer of both Tam (P<0.01) and 4OHT (P<0.05), but not End, exhibited a higher potency at hCB2Rs when compared to the respective E-isomer (Table 2). E- and Z-Tam were more efficacious hCB2R inverse agonists than the known full CB2R inverse agonist AM-630 (e.g., E_MAX_ values of 374–464%, relative to 214%, respectively). However, since complete sigmoidal curves with saturable effects for SERM modulation of adenylyl cyclase activity in CHO-hCB2 cells could not be obtained (likely due to poor SERM solubility at higher concentrations), such direct comparisons are tenuous, as EC_50_ and E_MAX_ values presented are only approximate. To confirm that the effects of SERMs on adenylyl cyclase activity observed result from specific interaction with hCBRs, similar studies were conducted in CHO-hMOR cells, devoid of hCBRs (Fig 5A–5C; diagonal bars). In agreement with data presented in the concentration-effect curves (Fig 4), a near receptor saturating concentration (10 μM) of both the E- and Z-isomers of Tam (Fig 5A), 4OHT (Fig 5B) and End (Fig 5C) produce from 180 to 300% increase in cAMP levels in CHO-hCB2 (open bars), but not CHO-hMOR (diagonal bars) cells. Finally, to provide additional support that the SERMs examined modulate adenylyl cyclase activity via hCB2Rs, CHO-hCB2 cells were treated overnight with pertussis toxin (100 ng/ml) to eliminate the ability of hCB2Rs to activate G_i/o_*-*proteins [39]. As anticipated for receptors producing effects via G_i/o_*-*proteins, such as CB2Rs, overnight treatment with pertussis toxin totally eliminated the ability of SERMs to alter cAMP levels (Fig 5A–5C; open bars). Collectively, these results suggest that the E- and Z-isomers of Tam, 4OHT and End act as full hCB2R inverse agonists for modulation of both G-protein and adenylyl cyclase activity.

**Table 2:** Modulation of adenylyl cyclase activity by SERM isomers in CHO-hCB2 cells.

Drug	Intracellular [^3^H]cAMP			
	EC_50_ (μM)	pEC_50_	E_MAX_ (%)	N
**AM-630**	**0.398**	**6.40 ± 0.096**	**214 ± 6.5**	**4**
**E-Tam**	**38.0**	**4.42 ± 0.081**^**a**^^,^^**^	**464 ± 48**^**a**^^,^ ^**††**^	**3**
**Z-Tam**	**8.71**	**5.06 ± 0.131** ^**b**^	**374 ± 24**^**a**^^,^^**b**^^,^ ^**†††**^	**3**
**E-4OHT**	**9.12**	**5.04 ± 0.082** ^**b**^^,^^*^	**352 ± 52**^**a**^^,^^**b**^	**3**
**Z-4OHT**	**4.90**	**5.31 ± 0.093** ^**b**^	**323 ± 37**^**a**^^,^^**b**^	**3**
**E-End**	**10.72**	**4.97 ± 0.123** ^**b**^	**239 ± 22**^**b**^	**6**
**Z-End**	**11.48**	**4.94 ± 0.059** ^**b**^	**259 ± 18**^**b**^	**6**

^a,b^pEC_50_ and E_MAX_ values designated by different letters are significantly different from values within the same column (AM-630 not included in analysis); P<0.05, one-way ANOVA, Tukey *Post-hoc* test.

*,**pEC_50_ values of the E-isomer are significantly different from the Z-isomer of the same compound within the same column; P<0.05, 0.01, student’s *t*-test.

^††,†††^ E_MAX_ values are significantly different from the full hCB2 inverse agonist AM-630; P<0.01, 0.001, student’s *t*-test.

### SERM isomers act as surmountable and insurmountable antagonists at hCB1Rs

**Fig 6:**
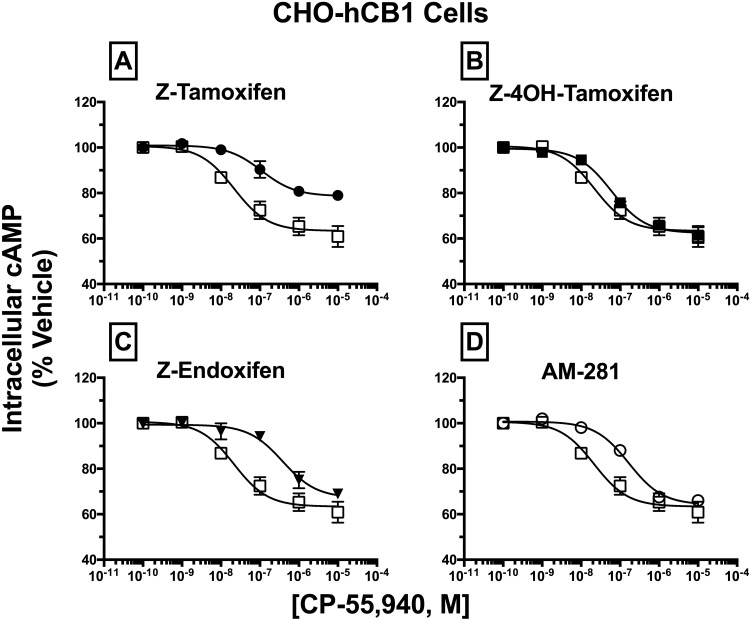
Antagonism of CP-55,940 inhibition of forskolin-stimulated AC activity by SERM isomers in intact CHO-hCB1 cells. CHO-hCB1 cells were pre-incubated for 30 min with receptor saturating concentrations of individual SERMs and were subsequently co-incubated for 7 min with increasing concentrations of CP-55,940. Measurements of CP-55,940 effects alone on potency (IC_50_) and efficacy (E_MAX_) of intracellular cAMP were obtained and were compared to the shifts in IC_50_ and E_MAX_ values observed in [A-D]. All IC_50_, EC_50_, and K_B_ values (mean ± SEM) were derived from non-linear regression analysis of the curves shown in [A-D] and are presented in Table 3 with statistical analysis. Open squares represent the concentration-effect curve for CP-55,940 alone, while filled symbols represent the action of CP-55,940 in the presence of the SERM indicated [A-C] or the selective CB1R inverse agonist AM-281 [D].

**Fig 7:**
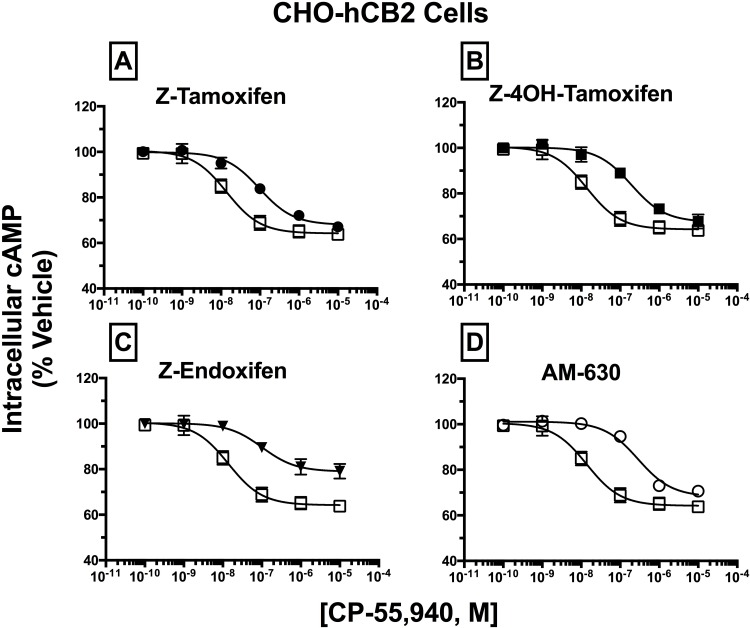
Antagonism of CP-55,940 inhibition of forskolin-stimulated AC activity by SERM isomers in intact CHO-hCB2 cells. CHO-hCB2 cells were pre-incubated for 30 min with receptor saturating concentrations of individual SERMs and were co-incubated for 7 min with increasing concentrations of the agonist CP-55,940. Measurements of CP-55,940 effects alone on potency (IC_50_) and efficacy (E_MAX_) of intracellular cAMP were obtained and were compared to the shifts in IC_50_ and E_MAX_ values observed in [A-D]. All IC_50_, EC_50_, and K_B_ values (mean ± SEM) were derived from non-linear regression analysis of the curves shown in [A-D] and are presented in Table 4 with statistical analysis. Open squares represent the concentration-effect curve for CP-55,940 alone, while filled symbols represent the action of CP-55,940 in the presence of the SERM indicated [A-C] or the selective CB2R inverse agonist AM-630 [D].

To demonstrate potential pharmacological relevance for this novel class of CBR ligands, studies were next conducted to determine whether SERMs act as CBR inverse agonists/antagonists when co-incubated with agonists (Figs 6 and 7). These experiments were limited to co-incubation of CBR agonists with only the Z-isomer of the SERMs, because Z-Tam is the isomer of Tam that is used therapeutically [23, 40–42]. To examine action at hCB1 receptors (Fig 6; Table 3), full concentration-effect curves for the known full CB1/CB2 agonist CP-55,940 were conducted in the absence (open symbols) and presence (closed symbols) of a receptor saturating concentration (e.g., >10 times K_i_; Table 1) of Z-Tam (Fig 6A; 30 μM), Z-4OHT (Fig 6B;10 μM) or Z-End (Fig 6C; 30 μM). To quantify the effect of antagonist co-incubation, measures of potency (IC_50_) and efficacy (I_MAX_) of CP-55,940 obtained from the concentration-effect curves were compared between treatments (Table 3). When co-incubation produced surmountable antagonism, antagonist dissociation constants (K_b_) were calculated [43]. K_b_ values were not determined when co-incubation resulted in insurmountable antagonism, as this violates the assumption of competitive antagonism required for K_b_ calculation. IC_50_ and K_b_ values were converted to pIC_50_ and pK_b_ values (pIC_50_ = -Log[IC_50_] or pK_b_ = -Log[K_b_], respectively) so that parametric tests could be used for statistical comparisons. In the absence of any antagonist, CP-55,940 reduced intracellular cAMP levels in CHO-hCB1 cells in a concentration-dependent manner, with a potency (IC_50_) of 20.0 nM and efficacy (I_MAX_) of 36.7%. As anticipated for the known competitive CB1R inverse agonist/antagonist AM-281, co-incubation resulted in a parallel rightward-shift in the concentration-effect curve for CP-55,940. Specifically, AM-281 produced a greater than 8-fold decrease in potency (IC_50_) of CP-55,940 (P<0.01) with no change in efficacy (I_MAX_) (e.g., surmountable antagonism; Fig 6D), resulting in a K_b_ value of 154 nM (Table 3). Co-incubation with Z-4OHT (Fig 6B) and Z-End (Fig 6C) produced similar parallel rightward-shifts (P<0.01, 0.01, respectively) with surmountable antagonism, resulting in K_b_ values of 4705 and 2514 nM, respectively. Very interestingly, although co-incubation with Z-Tam (Fig 6A) also resulted in over a 3-fold shift-to-the-right in the potency (IC_50_) of CP-55,940 (P<0.05), the resulting antagonism was insurmountable, with CP-55,940 producing maximal inhibition (I_MAX_) levels of only 23.0%, when compared to 36.7% in the absence of any antagonist (P<0.05). In agreement with results presented thus far for receptor affinity and intrinsic activity, these data confirm that SERMs act in isomer-specific manners at hCB1Rs to produce both surmountable and insurmountable antagonism of agonist-mediated inhibition of adenylyl cyclase activity.

**Table 3:** SERM isomer antagonism of CP-55,940 inhibition of AC-activity in intact CHO-hCB1 cells.

Drug	Intracellular [^3^H]cAMP						
	Pre-Incubation	IC_50_ (nM)	pIC_50_	I_MAX_ (%)	K_b_ (nM)	pK_b_	N
**CP-55,940**		**20.0**	**7.70 ± 0.144**	**36.7 ± 4.6**^**a**^			**3**
**+AM-281**	**1 μM**	**166**	6.78 ± 0.053^**^	**35.7 ± 0.9**^**a**^	**155**	**6.81 ± 0.061**	**3**
**+Z-Tam**	**30 μM**	**64.6**	7.19 ± 0.091^*^	**23.0 ± 0.5**^**b**^	**N/D**	**N/D**	**4**
**+Z-4OHT**	**10 μM**	**70.8**	7.15 ± 0.049^**^	**38 ± 2.8**^**a**^	**4677**	**5.33 ± 0.071**	**5**
**+Z-End**	**30 μM**	**295**	6.53 ± 0.157^**^	**35 ± 1.8**^**a**^	**2570**	**5.59 ± 0.170**	**3**

N/D represents values not determined.

*,**pIC_50_ values are significantly different from the pIC_50_ value for CP-55,940; P<0.05, 0.01, student’s *t*-test.

^a,b^I_MAX_ values designated by different letters are significantly different from values within the same column; P<0.05, one-way ANOVA, Tukey *Post-hoc* test.

### SERM isomers act as surmountable and insurmountable antagonists at hCB2Rs

Similar studies were conducted to determine the effect of SERM co-incubation on the potency and efficacy of agonists acting at hCB2Rs (Fig 7; Table 4). Full concentration-effect curves for the known full CB1/CB2 agonist CP-55,940 were conducted in the absence (open symbols) and presence (closed symbols) of a receptor saturating concentration (e.g., >10 times K_i_; Table 1) of Z-Tam (Fig 7A; 10 μM), Z-4OHT (Fig 7B;10 μM) or Z-End (Fig 7C; 30 μM). Co-incubation with the known competitive CB2R inverse agonist/antagonist AM-630 (Fig 7D) produced a parallel rightward-shift in the concentration-effect curve for CP-55,940 (P<0.001), resulting in a K_b_ value of 55.4 nM (Table 4). Similar parallel rightward-shifts with surmountable antagonism were produced by Z-Tam (Fig 7A) and Z-4OHT (Fig 7B) co-incubation (P<0.01, 0.001, respectively), with K_b_ values of 1787 and 856 nM, respectively. As observed for Z-Tam at hCB1Rs, co-incubation with Z-End (Fig 7C) produced over an 8-fold shift-to-the-right in the potency (IC_50_) of CP-55,940 (P<0.01). However, the resulting antagonism of CP-55,940 was insurmountable, with a maximal inhibition (I_MAX_) of only 20.7.%, as opposed to 35.6% in the absence of Z-End (P<0.05). Consistent with studies presented for receptor affinity and intrinsic activity, these results validate that SERMs are hCB2R antagonists, producing both surmountable and insurmountable antagonism in an isomer-specific manner.

**Table 4:** SERM isomer antagonism of CP-55,940 inhibition of AC-activity in intact CHO-hCB2 cells.

Drug	Intracellular [^3^H]cAMP						
	Pre-Incubation	IC_50_ (nM)	pIC_50_	I_MAX_ (%)	K_b_ (nM)	pK_b_	N
**CP-55,940**		**14.1**	**7.85 ± 0.144**	**35.6 ± 2.2**^**a**^			**3**
**+AM-630**	**1 μM**	**288**	6.54 ± 0.060^***^	**31.7 ± 0.3**^**a**^	**55.0**	**7.26 ± 0.064**	**3**
**+Z-Tam**	**10 μM**	**100**	7.00 ± 0.064^**^	**32.3 ± 0.7**^**a**^	**1778**	**5.75 ± 0.064**^**x**^	**3**
**+Z-4OHT**	**10 μM**	**191**	6.72 ± 0.014^***^	**32.7 ± 2.0**^**a**^	**851**	**6.02 ± 0.015**	**3**
**+Z-End**	**30 μM**	**120**	6.92 ± 0.126^**^	**20.7 ± 3.5**^**b**^	**N/D**	**N/D**	**3**

N/D represents values not determined.

**,***pIC_50_ values are significantly different from the pIC_50_ value for CP-55,940; P<0.01, 0.001, student’s *t*-test.

^a,b^I_MAX_ values designated by different letters are significantly different from values within the same column; P<0.05, one-way ANOVA, Tukey *Post-hoc* test.

^x^pK_b_ value is significantly different from Z-4OHT; P<0.05, student’s *t*-test.

## Discussion and Conclusions

The studies presented here demonstrate that in addition to differential binding affinity for ERs, the E and Z-isomers of Tam, 4OHT and End also exhibit distinct affinity and selectivity for CBRs. Specifically, it was shown that Z-4OHT, but not Z-Tam or Z-End, exhibits higher affinity for both CB1 and CB2Rs relative to the E-isomer. Furthermore, both Tam isomers show higher affinity for CB1Rs, while Z-End is relatively CB1R-selective and E- and Z-4OHT are non-selective. Although the 100-fold differences in affinity between the E- [41] and Z-isomers [23, 42] of SERMs for ERs were not reflected here for CBRs, our studies nevertheless suggest that this novel structural scaffold might be employed for future drug development of selective, high affinity CB1 and CB2R ligands.

Similar to affinity for CBRs, the SERM isomers examined also exhibit significant differences in intrinsic activity at CBRs, as reflected by modulation of G-protein and AC activity. Constitutively active CBRs or binding of agonists to CBRs results in activation of Gi-proteins that then proceed to regulate several intracellular effectors [44, 45]. Consistent with actions of inverse agonists that reduce constitutive activity of hCB1Rs, all SERMs examined (except E-4OHT) significantly reduce basal G-protein activity, with the Z-isomers of Tam and 4OHT exhibiting higher efficacy when compared to the corresponding E-isomers. All SERMs tested similarly act as inverse agonists at hCB2Rs, reducing basal G-protein activity, with Z-Tam also acting as a more efficacious inverse agonist at hCB2Rs than E-Tam. Because Z-Tam slightly reduces basal G-protein activity in CHO-hMOR membranes devoid of CBRs (see Fig 3C), it is possible that the greater efficacy observed for Z- relative to E-Tam at hCB1 and hCB2Rs might result, in part, from actions independent of CBRs. However, such potential confounding effects cannot explain the greater efficacy observed for Z-4OHT at hCB1Rs. Most interestingly, in this assay E-4OHT acts as a neutral antagonist at hCB1Rs, while Z-4OHT exhibits actions consistent with that of a full hCB1R inverse agonist. The apparent neutral antagonist activity of E-4OHT at hCB1Rs is of particular significance, given the recent push to develop hCB1R neutral antagonists devoid of negative intrinsic activity [46], to treat a variety of disease states [47] with reduced adverse effects [48].

Constitutively active CBRs stimulate G_i/o_-proteins that inhibit AC activity, ultimately leading to reduced levels of intracellular cAMP in intact cells [49]. Therefore, inverse agonists that reduce constitutive activity of hCBRs would be anticipated to not only reduce basal G-protein activity, but also increase levels of intracellular cAMP in intact cells stably expressing these receptors. Surprisingly, the well characterized CB1R inverse agonist AM281 [50] did not alter basal intracellular cAMP levels in intact CHO-hCB1 cells (see Fig 4), indicating that regulation of AC activity by constitutively active hCB1Rs is unfortunately below the level of detection required to quantify potential inverse agonist activity of SERMs in these cells. However, in CHO-hCB2 cells, the hCB2 inverse agonist AM630 [51] and all SERMs examined produce concentration-dependent increases in cAMP levels, consistent with actions as inverse agonists. Furthermore, the Z-isomers of Tam and 4OHT are more potent relative to the respective E-isomers, which is in agreement with a similar rank order of affinity for hCB2Rs, and parallels activity of these isomers at ERs [41]. Most importantly, the E- and Z-isomers of both Tam and 4OHT, but not End, also efficaciously increase intracellular cAMP levels. These observations are significant, given the proposed development of CB2 inverse agonists for potential therapeutic use as immunomodulators [52, 53]. Furthermore, since SERMs have been used clinically by thousands of patients, for years at a time to treat cancer [54] and osteoporosis [55] with few adverse effects, drugs in this class might easily and quickly be repurposed for use in diseases shown in preclinical studies to potentially benefit from use of CB2R inverse agonists. For example, pain resulting from chronic inflammation in mice has been shown to respond well to treatment with CB2R inverse agonists [52].

In addition to establishing that SERMs act as inverse agonists at hCBRs when administered alone, the present study also importantly determined if SERMs act as antagonists when co-incubated with cannabinoid agonists. Antagonist studies were limited to examination of only the Z-isomers of Tam, 4OHT and End, because the Z-isomers of SERMs are used clinically [56] due to higher affinity and potency at ERs relative to E-isomers [42]. Very interestingly, co-incubation with SERMs produces both surmountable and insurmountable antagonism of AC-inhibition mediated by the CB1/CB2 agonist CP-55,940 at both hCB1 and hCB2Rs. Concerning hCB1Rs, co-incubation with Z-4OHT and Z-End produces surmountable antagonism, reflected by parallel rightward shifts in the agonist concentration-effect curves, consistent with actions of competitive antagonists [57]. In contrast, although co-incubation with Z-Tam results in an over 3-fold shift-to-the-right in the agonist concentration-effect curve, the antagonism is insurmountable, as indicated by a significant reduction in agonist efficacy. Although both types of antagonism are also observed by SERMs acting at hCB2Rs, for this receptor Z-Tam and Z-4OHT produce surmountable, while Z-End acts as an insurmountable antagonist. Additional evidence indicating that the observed surmountable antagonism produced by SERM co-incubation occurs specifically via CBRs is provided by observation that the rank order of K_b_ (antagonist dissociation constant) and K_i_ (receptor affinity) values for SERMs acting at hCB1 and hCB2Rs is identical. For example, the rank order of both K_b_ and K_i_ values for SERMs acting at hCB1Rs is AM281 >> Z-End > Z-4OHT, while the rank order for these compounds at hCB2Rs is AM630 >> Z-4OHT > Z-Tam. K_b_ values for insurmountable antagonists could not be determined because competitive antagonism is an assumption required for calculation of this constant [58].

Future experiments will be required to fully characterize the underlying mechanisms responsible the insurmountable antagonism produced by the SERMs reported here [59, 60]. However, it is likely that these compounds either irreversibly bind to [61], or interact with an allosteric site distinct from the orthosteric binding site within [62], CBRs. The most well characterized cannabinoids to date interact with the orthosteric binding site within CBRs, the site to which endogenously produced endocannabinoids bind. As a means to reduce adverse effects produced by conventional cannabinoid ligands, compounds are being developed that instead bind to allosteric sites on both CB1 and CB2Rs (*e*.*g*., allosteric modulators), to modulate the signaling properties of concurrently administered synthetic cannabinoids or endocannabinoids released due to injury or disease [60]. Allosteric modulators devoid of intrinsic activity when given alone would be ideal, given that these compounds would neither activate nor inhibit basal receptor activity in absence of an orthosteric agonist [63]. Both SERMs identified in the present study as potential allosteric modulators due to insurmountable antagonist properties, unfortunately also act as inverse agonists at CBRs. However, it is possible that future molecular modeling and structure-activity-relationship (SAR) studies utilizing the pharmacological properties of SERMs reported here, coupled with study of related compounds, may ultimately lead to development of a class of novel allosteric modulators of CBRs, devoid of intrinsic activity, that exhibit reduced adverse effects when used clinically.

Interestingly, it should be noted that in this study, and as reported previously [17, 18], special assay conditions were needed to observe optimal CBR antagonism by SERMs. For example, assays were conducted at room temperature with a 30 min SERM pre-incubation period, followed by agonist exposure for 7 min. Although not determined here, it is possible that SERMs bind less tightly to CBRs when compared to CP-55,940, and thus a lower assay temperature improves thermodynamic conditions that favor optimal SERM binding [64].

Another important question raised by the data presented here involves the potential therapeutic relevance of compounds, such as SERMs, which exhibit affinities for molecular targets in the low micromolar range. In other words, it might be questioned whether SERM concentrations can be attained in the serum and/or tissues sufficient to elicit physiological effects via CBRs. Chronic administration of Tam and 4OHT has importantly been reported to reach high nanomolar concentrations in serum [40], and accumulate in brain and breast tissue to levels in the micromolar range [65, 66]. Therefore, repurposing clinically available SERMs for use as CBR inverse agonists to treat diseases resulting from an overactive endocannabinoid system may have an exciting potential therapeutic relevance (see following).

Recent studies have shown that CBR inverse agonists may have potential therapeutic relevance in many disease states, including cancer, osteoporosis, alcoholism, liver cirrhosis and cardiovascular toxicity [52, 67–69]. Specifically, cannabinoid agonists including Δ^9^-THC and CP-55,940 have been studied for decades and shown to induce cancer cell death, inhibit angiogenesis and block tumor invasion and metastasis of numerous cancer cell types [70]. Interestingly, the CB1 inverse agonist rimonabant also inhibits proliferation of MDA-MB-231 breast cancer cells by inhibition of ERK1/2 activity and blunting CB1R associated lipid raft trafficking [71]. Rimonabant modulates apoptosis of U251 glioma cells by inducing cell cycle arrest in the G_1_ phase and blocking TGF-β1 secretion via STAT3 inhibition [72]. In addition to such potential new therapeutic uses, the inverse agonist actions of Tam and its metabolites at CBRs might also contribute to adverse effects associated with chronic Tam usage. For example, similar to side effects observed with Tam in humans [73], use of CBR inverse agonists increases bone mineralization, nociception sensitivity and may result in depression [74–77]. Taken as a whole, data presented here suggest that future studies are needed to more precisely define the role of CBRs in both the therapeutic and adverse effects of Tam.

## Conclusions

In summation, results from our study demonstrate that the SERMs Tam, 4OHT and End elicit ER-independent actions via CBRs in an isomer-specific manner. For example, Z-4OHT, but not Z-Tam or Z-End, exhibits higher affinity for both CB1 and CB2Rs relative to the E-isomer. Although the Z- and E-isomers of Tam and 4OHT exhibit slightly higher affinity for CB2Rs, both End isomers were relatively CB1R-selective. When functional assays evaluating G-protein and AC-activity are examined, all isomers act as full CB1 and CB2R inverse agonists. Both Z-Tam and Z-End exhibit characteristics of insurmountable antagonism at CB1 and CB2Rs, respectively. Collectively, these results suggest that Tam might serve as a novel molecular scaffold to develop safe and therapeutically useful drugs for treatment of a variety of diseases mediated via CBRs.

## Abbreviations

SERM: selective estrogen receptor modulator
E: *cis*
Z: *trans*
Tam: tamoxifen
4OHT: 4-hydroxytamoxifen
End: 4-hydroxy-N-desmethyltamoxifen
AC: adenylyl cyclase
BSA: bovine serum albumin
CB1R: cannabinoid type-1 receptor
CB2R: cannabinoid type-2 receptor
CHO-hCB1: CHO cells expressing human CB1Rs
CHO-hCB2: CHO cells expressing human CB2Rs
PTX: pertussis toxin
Δ^9^-THC: delta-9-tetrahydrocannabinol
GDP: guanosine diphosphate
GTPγS: guanosine 5ano-[gamma-thio]triphosphate
GPCR: G-protein coupled receptor
